# Natural variation in acyl editing is a determinant of seed storage oil composition

**DOI:** 10.1038/s41598-018-35136-6

**Published:** 2018-11-26

**Authors:** Guillaume N. Menard, Fiona M. Bryant, Amélie A. Kelly, Christian P. Craddock, Irene Lavagi, Keywan Hassani-Pak, Smita Kurup, Peter J. Eastmond

**Affiliations:** 1 Department of Plant Science, Rothamsted Research, Harpenden, Hertfordshire AL5 2JQ UK; 2 Georg-August-University, Albrecht-von-Haller-Institute for Plant Sciences, Justus-von-Liebig Weg 11, 37077 Göttingen, Germany; 3 Mt. San Jacinto College, Menifee Valley Campus, 28237 La Piedra Road, Menifee, CA 92584 USA; 4 Department of Microbiology and Plant Pathology, University of California Riverside, Riverside, CA 92521 USA

## Abstract

Seeds exhibit wide variation in the fatty acid composition of their storage oil. However, the genetic basis of this variation is only partially understood. Here we have used a multi-parent advanced generation inter-cross (MAGIC) population to study the genetic control of fatty acid chain length in *Arabidopsis thaliana* seed oil. We mapped four quantitative trait loci (QTL) for the quantity of the major very long chain fatty acid species 11-eicosenoic acid (20:1), using multiple QTL modelling. Surprisingly, the main-effect QTL does not coincide with *FATTY ACID ELONGASE* *1* and a parallel genome wide association study suggested that *LYSOPHOSPHATIDYLCHOLINE ACYLTRANSFERASE 2* (*LPCAT2*) is a candidate for this QTL. Regression analysis also suggested that *LPCAT2* expression and 20:1 content in seeds of the 19 MAGIC founder accessions are related. LPCAT is a key component of the Lands cycle; an acyl editing pathway that enables acyl-exchange between the acyl-Coenzyme A and phosphatidylcholine precursor pools used for microsomal fatty acid elongation and desaturation, respectively. We Mendelianised the main-effect QTL using biparental chromosome segment substitution lines and carried out complementation tests to show that a single *cis*-acting polymorphism in the *LPCAT2* promoter causes the variation in seed 20:1 content, by altering the *LPCAT2* expression level and total LPCAT activity in developing siliques. Our work establishes that oilseed species exhibit natural variation in the enzymic capacity for acyl editing and this contributes to the genetic control of storage oil composition.

## Introduction

Seed maturation is associated with the deposition of storage reserves, such as oil (triacylglycerol), carbohydrates and proteins^1^. The most common reserve is oil, which can account for up to ~70% of seed weight in some species^2^. The physiological role of storage oil is to provide a source of carbon to support post-germinative growth, thereby allowing seedling establishment and the completion of the plant’s life cycle^3^. However, seed storage oils also serve as a primary source of nutrition for humans and livestock, and provide renewable chemical feedstock for a variety of industrial applications^4^. The fatty acid composition of seed storage oils varies greatly^5^ and is believed to have adaptive significance^6^. For example, biogeographic studies indicate that the degree of fatty acid unsaturation has played a role in local adaptation to temperature on a micro- and a macroevolutionary scale^7,8^. Understanding how plants control seed storage oil content and fatty acid composition is of both strategic and fundamental interest^1,9^.

The model plant *Arabidopsis thaliana* has served as a powerful research tool to study many aspects of seed biology. Much of our molecular understanding of storage oil deposition in dicotyledonous seeds is founded on *Arabidopsis* mutant studies^1^. Forward and reverse genetic screens have identified the network of transcriptional master regulators that orchestrate the seed maturation program^10,11^, as well as many downstream components of the metabolic machinery that partitions imported sucrose into triacylglycerol (TAG)^1,9^. Much of the underpinning knowledge obtained from the study of *Arabidopsis* has also proved useful in understanding crop species, such as oilseed rape (*Brassica napus*), which is also a member of the Brassicaceae.

*Arabidopsis* has a wide geographical distribution and exhibits significant natural variation in seed TAG content and composition^12,13^. Several studies have used recombinant inbred populations derived from bi-parent crosses to map quantitative trait loci (QTL) controlling oil composition^6,14–16^. The power to detect QTL with this method is high because the minor allele frequency (*p*) is ~0.5, but mapping resolution is often relatively poor (~5 to 20 cM)^17^. Branham *et al*., (2016) have also performed a genome-wide association study (GWAS) of oil composition using ~200,000 sequence variants in a panel of ~400 accessions^18^. The mapping resolution of this approach is superior because linkage disequilibrium decays rapidly in natural accessions^19^. However, QTL discovery rate can be lower because *p* is <0.5 for many alleles^20^ and marker coverage is usually incomplete^21^. Complex population structure^20^ and allelic heterogeneity at causal loci^22^ can also lead to spurious and ghost associations. Although previous studies have identified many genomic regions that are associated with seed TAG composition^6,14,16,18^, only one QTL has ever been fine-mapped and the causal sequence variant determined^15^.

The development of multi-parent advanced generation inter-cross (MAGIC) populations allows the complementary use of both linkage and association methodologies, without any confounding caused by population structure^23^. Kover *et al*., (2009) created a large *Arabidopsis* MAGIC population that encompasses the genetic variation within 19 founder accessions and consists of >500 recombinant inbred lines (RILs)^24^. Both the founder accessions and the RILs, have been sequenced providing comprehensive marker coverage, consisting of ~3 million individual sequence variants^25,26^. *Arabidopsis* seed TAG is primarily composed of polyunsaturated fatty acids (PUFAs) and very long chain fatty acids (VLCFAs)^12,13^. We have recently used the MAGIC population to study the genetic control of fatty acid desaturation in seeds^27,28^. The aim of this study was to investigate the control of fatty acid elongation and to identify both QTL and their underlying causal sequence variants.

## Results

### *FAE1* is not the major determinant of seed 20:1 content in the MAGIC population

*Arabidopsis thaliana* seed TAG contains a high proportion of VLCFAs, of which 11-eicosenoic acid (20:1) is the predominant form^12,13^. Kover *et al*., (2009) previously selected 19 *Arabidopsis* founder accessions, representing a wide range of genotypic and phenotypic diversity, to construct a MAGIC population^24^. To investigate whether this population would contain significant variation in 20:1 content, we first analysed the fatty acid composition of seeds from the 19 founder accessions^28^. The 20:1 content ranged between 18.2 ± 0.2 and 22.1 ± 0.3 mol% (*n* = 5, s.e.m.). We therefore analysed the fatty acid composition of 427 RILs from the MAGIC population, that were grown as three biological replicates in a random block design experiment^28^. The broad-sense heritability (H^2^) for 20:1 was high (0.85) and line averages for the RILs ranged between 16.7 ± 0.3 and 22.9 ± 0.1 mol% (*n* = 3, s.e.m.).

We used the seed 20:1 content line averages to carry out both QTL analysis and GWAS, exploiting genomic resources and software tools developed for the MAGIC population by Richard Mott and colleagues^24–26^. Using multiple QTL modelling, we identified four 20:1 QTL, with a genome-wide *P* value < 0.01 (Supplementary Table 1). 20:1q2 accounted for most of the phenotypic variation in the trait and is situated on Chromosome 1 at ~23.3 Mb. The 90% confidence interval (CI) for this QTL is ~0.8 Mb^24^. The other three more minor QTL (20:1q1, 3 and 4) are situated on Chromosomes 1, 4 and 5 at ~0.6, ~17.4 and ~11.8 Mb, respectively (Supplementary Table 1). The 90% CI for 20:1q3 corresponds approximately to the location of *FATTY ACID ELONGASE* *1* (*FAE* *1)*, which is situated at ~16.5 Mb on Chromosome 4. *FAE* *1* encodes the β-ketoacyl-Coenzyme A synthase activity of the fatty acid elongase complex in developing seeds^29^ and has previously been shown to be a major-effect QTL that underlies variation in seed VLCFA / 20:1 content among several *Arabidopsis* accessions^6,15,16^.

### *LPCAT2* is a candidate for the main-effect QTL for 20:1 content in the MAGIC population

**Figure 1:**
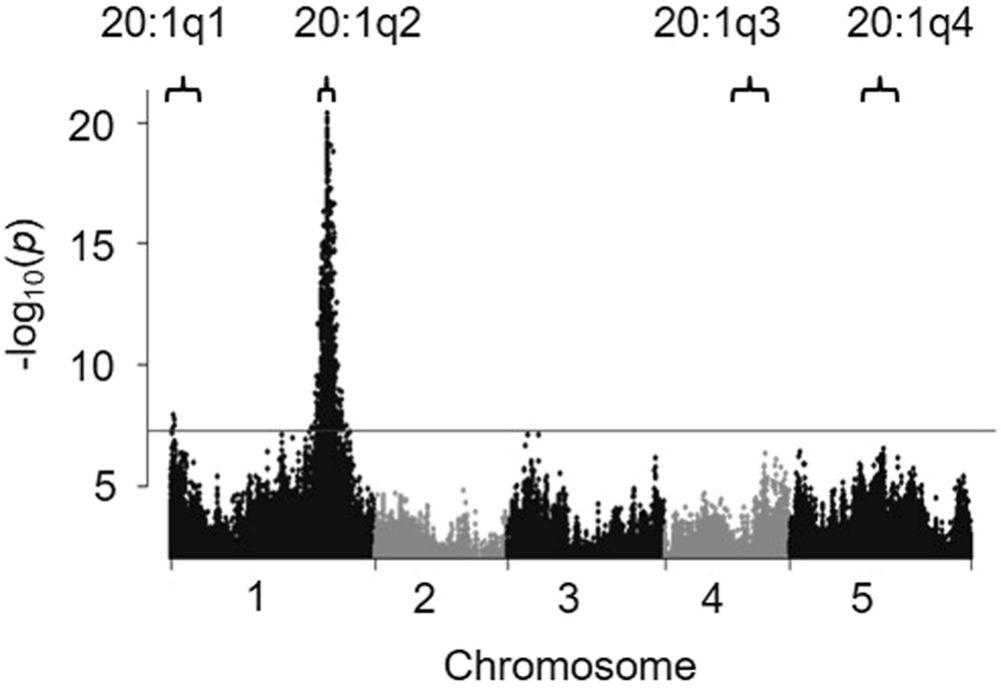
Manhattan plot showing association of ~3 million individual sequence variants with seed 20:1 content in the MAGIC population. Individual plants for 427 RILs were grown in three randomised blocks (*n* = 3) in the glasshouse and the seed for each plant were harvested separately and analysed. GWAS was performed using the ‘magic_src_v4.0.tar.gz’ package. The 90% CIs for corresponding 20:1 QTL are bracketed and the grey line marks the genome-wide significance threshold.

In parallel to the QTL analysis, we also performed GWAS to identify individual polymorphisms associated with seed 20:1 content (Fig. 1). This analysis used all ~3 million sequence variants within the imputed genomes of the MAGIC RILs^25^. Polymorphisms with a –log_10_(*p*) score above the genome-wide significance threshold were detected only within the 90% CIs for 20:1q1 and 20:1q2 (Fig. 1). The polymorphisms were ranked by –log_10_(*p*) score and genes that map within 1 kb up or downstream were identified and listed, together with their gene names/descriptions from The Institute for Genome Research (Supplementary Dataset 1). The highest-ranking polymorphisms within the 20:1q2 CI are all confined to a ~70 kb region and lie within 1 kb of 14 genes (Supplementary Table 2). The function of these genes was investigated by searching relevant databases such as ARALIP^9^, the *Arabidopsis* Information Resource (https://www.Arabidopsis.org/) and KnetMiner^30^. The strongest candidate amongst the 14 genes is *LYSOPHOSPHATIDYLCHOLINE ACYLTRANSFERASE 2* (*LPCAT2*), based on prior knowledge of gene function^9,31,32^.

LPCAT2 is the predominant LPCAT isoform in developing *Arabidopsis* seeds and catalyses the esterification of 1-lysophospatidylcholine (1-LPC) with acyl-Coenzyme A (acyl-CoA) to form phosphatidylcholine (PC)^31–33^. This reversible reaction^34,35^ is a key component of the Lands cycle, or acyl editing pathway, that allows newly synthesised fatty acids in the acyl-CoA pool to enter the *sn-2* position of PC for desaturation and subsequent assembly into TAG^31,32^. An alternative fate for fatty acids in the acyl-CoA pool is to undergo elongation to form VLCFAs such as 20:1^9^. Loss of *LPCAT2* function has been shown to increase seed 20:1 content, mainly at the expense of PUFAs^32,36^, while gain of function leads to a decrease in 20:1^32^. It is therefore conceivable that allelic variation in *LPCAT2* could underlie 20:1q2 and account for much of the phenotypic variation in seed 20:1 content within the MAGIC population.

### Seed 20:1 content in the MAGIC founder accessions is related to *LPCAT2* expression

**Figure 2:**
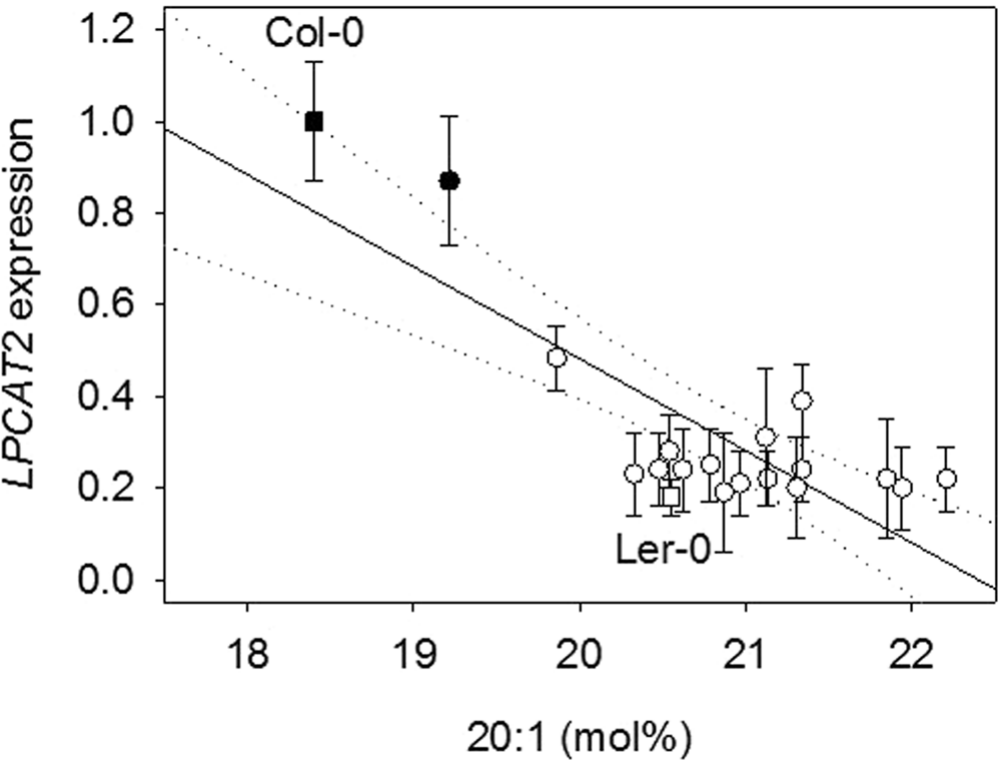
Relationship between *LPCAT2* expression and 20:1 content in seeds of the 19 founder accessions of the MAGIC population. Quantitative RT-PCR was performed on RNA from mature seeds of five separate plants of each genotype (*n* = 5) and values are normalised to Col-0 and expressed as the mean ± s.e.m. Seed 20:1 content is also the mean of measurements on five separate plants (*n* = 5) and all s.e.m. are < 0.3. Closed symbols are accessions that have the Col-0 *LPCAT2* haplotype (Supplementary Table 3) and square symbols highlight Col-0 and Ler-0. Regression analysis supports a negative linear relationship (R^2^ = 0.652; *P* < 0.05). The dotted line marks the 95% confidence interval.

Gan *et al*.^25^ have previously shown that transcript abundance data for the 19 founder accessions of the MAGIC population can be used to identify potential *cis*-acting variants associated with expression (i.e. *cis*-eQTL)^25^. The polymorphisms in *LPCAT2*, which are most strongly associated with seed 20:1 content, are located within the promoter (P1), first intron (P2) and 3′ intergenic region (P3 and P4) (Supplementary Table 3). If one or more of these polymorphisms cause the phenotypic variation in seed 20:1 content, they would most likely act by modifying the level of *LPCAT2* expression. We therefore used quantitative RT-PCR to measure *LPCAT2* transcript abundance in seeds from each of the 19 founder accessions. Linear regression analysis suggests that there is a significant negative relationship between *LPCAT2* transcript abundance and seed 20:1 content (R^2^ = 0.652; *P* < 0.05) (Fig. 2). Furthermore, *LPCAT2* expression level was also related to variation in the four polymorphisms at this locus (*P* < 0.05), which constitute a haplotype that distinguishes Colombia-0 (Col-0) and Rschew (Rsch-4) from the remaining 17 founder accessions (Fig. 2), including Landsberg *erecta* (Ler-0)^25,37^. *LPCAT2* transcript abundance was ~5-fold higher in Col-0 seeds than in Ler-0 (Fig. 2).

### The main-effect QTL 20:1q2 can be Mendelianised using biparental substitution lines

**Figure 3:**
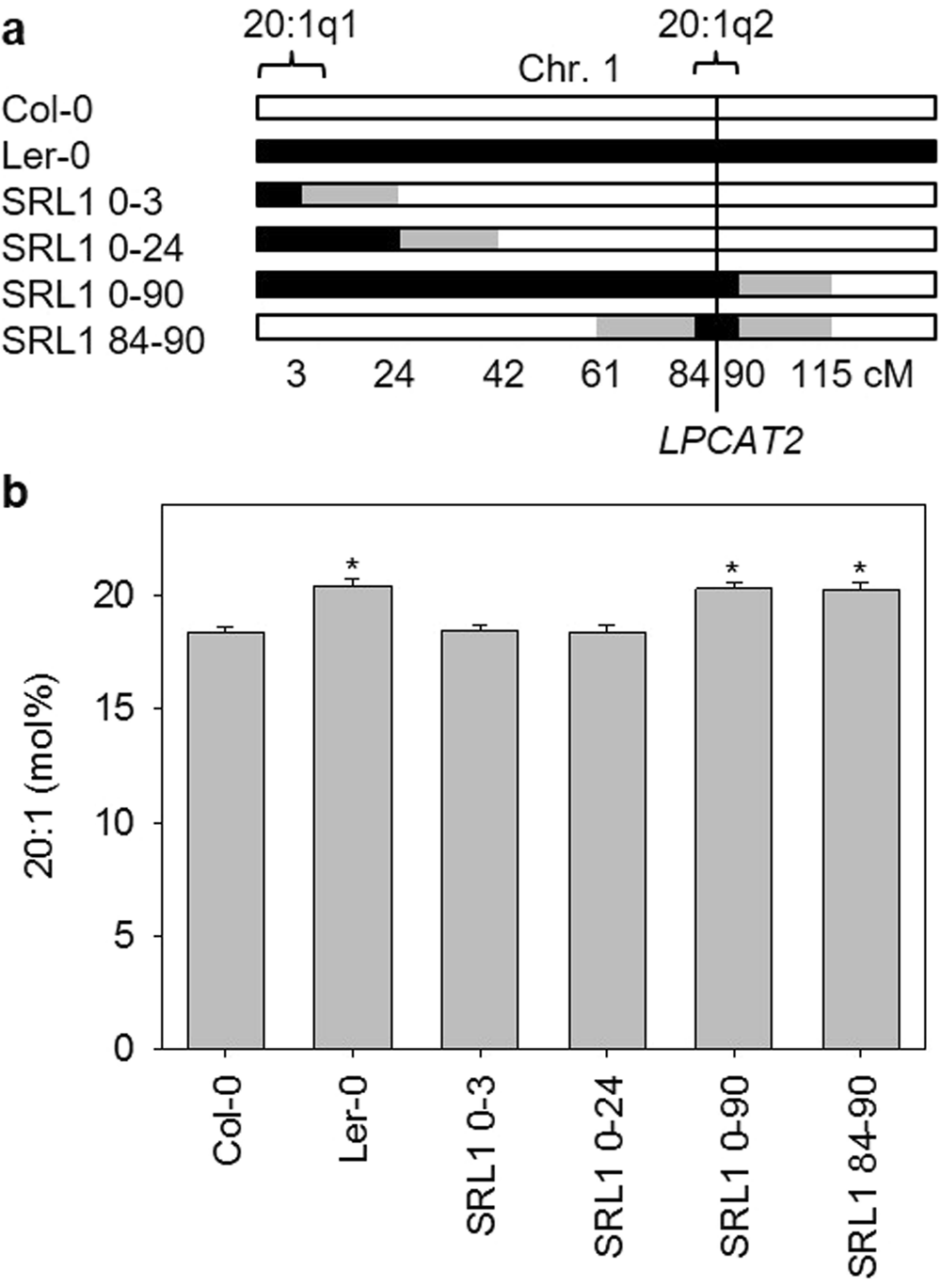
Mendelianisation of 20:1q2 using biparental substitution lines. (**a**) The positions of single Ler-0 introgressions on Chromosome 1^38^ are shown. Crossovers lie within the grey regions between markers. (**b**) The fatty acid composition of seeds from five separate plants of each genotype (*n* = 5) was determined and 20:1 content is the mean ± s.e.m. The asterisk denotes values that are significantly different (*P* > 0.05) from Col-0 (LSD-test).

To investigate whether allelic variation between Col-0 and Ler-0 can explain the main-effect QTL 20:1q2, we obtained three chromosome segment substitution lines with single Ler-0 introgressions on chromosome 1 in a Col-0 background^38^. SRL1 0–3, SRL1 0–24 and SRL1 0–84 contain introgressions between 0 and 3–24, 24–42 and 84–115 cM, respectively^38^. PCR-based genotyping using polymorphic markers showed that the Ler-0 introgression in SRL1 0–84 extends beyond ~90 cM (renamed SRL1 0–90) and therefore encompasses the 90% CIs of both 20:1q1 and 20:1q2 (Fig. 3a). Analysis of fatty acid composition showed that the 20:1 content of SRL1 0–90 seed is significantly higher than that of Col-0 (*P* > 0.05), whereas 20:1 content of SRL1 0–3 and SRL1 0–24 seed is not significantly different from Col-0 (Fig. 3b). These data are consistent with the presence of a 20:1q2 allele in Ler-0 that is hypomorphic to Col-0. We backcrossed SRL1 0–90 to Col-0 and used PCR-based polymorphic markers to obtain a new near isogenic line (NIL) from the F2 progeny. SRL1 84–90 contains a single Ler-0 introgression between 61–84 and 90–115 cM that encompasses 20:1q2, and not 20:1q1 (Fig. 3a). Fatty acid analysis of SRL1 84–90 seed confirmed that the 20:1 content is significantly higher than Col-0 (*P* > 0.05; Fig. 3b).

### A 20:1q2 NIL has a *lpcat2* seed fatty acid profile and reduced *LPCAT* expression and activity

**Figure 4:**
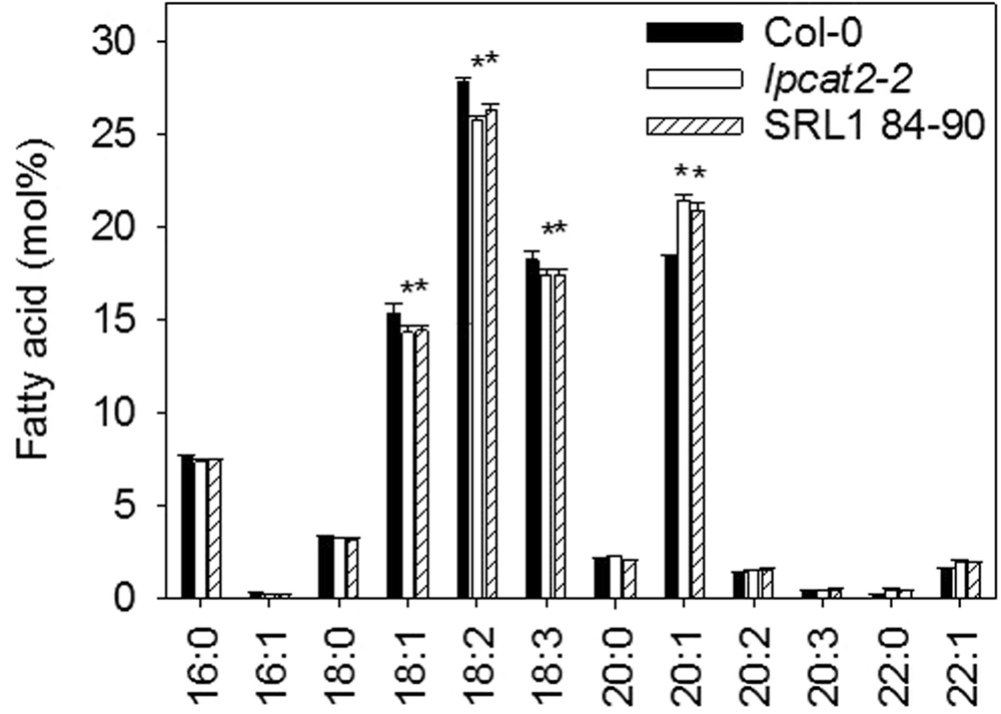
Seed fatty acid profile of Col-0, *lpcat2-2* and SRL1 84–90, carrying a single Ler-0 introgression in the region of 20:1q2. The fatty acid composition of seeds from five separate plants of each genotype (*n* = 5) was determined and values are the mean ± s.e.m. The asterisk denotes values that are significantly different (*P* > 0.05) from Col-0 (LSD-test).

**Figure 5:**
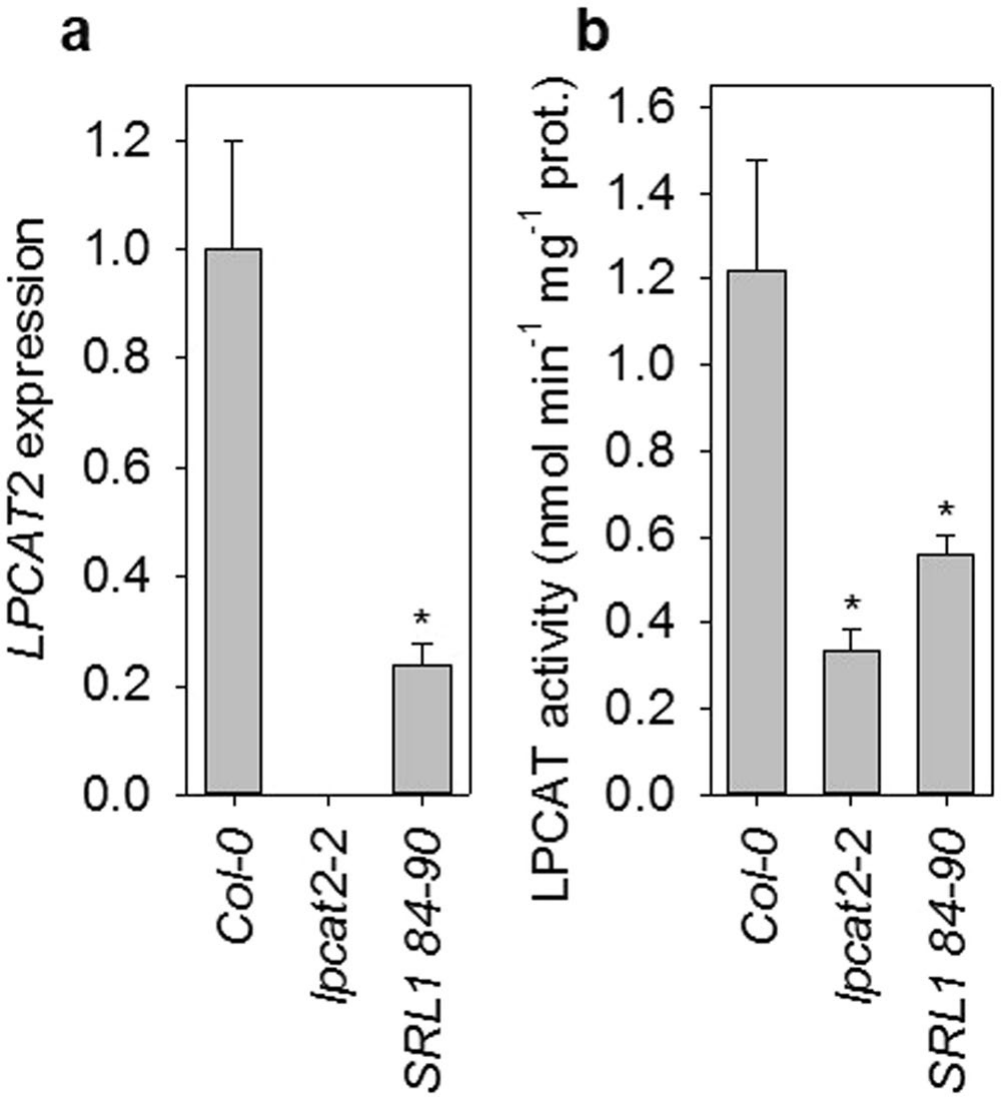
*LPCAT* expression and activity and Col-0, *lpcat2-2* and SRL1 84–90, carrying a single Ler-0 introgression in the region of 20:1q2. (**a**) Quantitative RT-PCR and (**b**) microsomal enzyme assays were performed on siliques containing seeds at stages 8 to 9 of development^60^. Values are the mean ± s.e.m. of measurements on three separate extracts (*n* = 3) and expression is normalised to that of Col-0. The asterisk denotes values that are significantly different (*P* > 0.05) from Col-0 (LSD-test).

20:1q2 maps to the location of *LPCAT2* (Fig. 1; Supplementary Table 2) and *lpcat2* null mutants in the same genetic background as SRL1 84–90 (Col-0) also have elevated 20:1 content^32,36^. Seed of *lpcat2* exhibit additional characteristic changes in fatty acid profile, including a reduction in PUFA content^32^, that results from reduced acyl-entry into the PC substrate pool for desaturation^31,32^. We therefore analysed the total fatty acid composition of SRL1 84–90 seed and found that oleic acid (18:1), linoleic acid (18:2) and linolenic acid (18:3) content are also significantly reduced (*P* > 0.05) relative to Col-0 (Fig. 4) and that the total fatty acid profile mirrors that of *lpcat2-2*^32^. To determine whether SRL1 84–90 is defective in LPCAT2 function we preformed quantitative RT-PCR analysis and microsomal enzyme activity on siliques containing developing seeds at curled to green cotyledons stage (i.e. stage 8 to 9)^32^. *LPCAT2* transcript abundance was reduced by ~80% in SRL1 84–90 siliques (Fig. 5a) and total LPCAT activity was reduced by ~60% (Fig. 5b). For comparison, *LPCAT2* transcripts were absent in *lpcat2-2* siliques and LPCAT activity was reduced by ~70% (Fig. 5b).

### A complementation test suggests 20:1q2 and *LPCAT2* are synonymous

**Figure 6:**
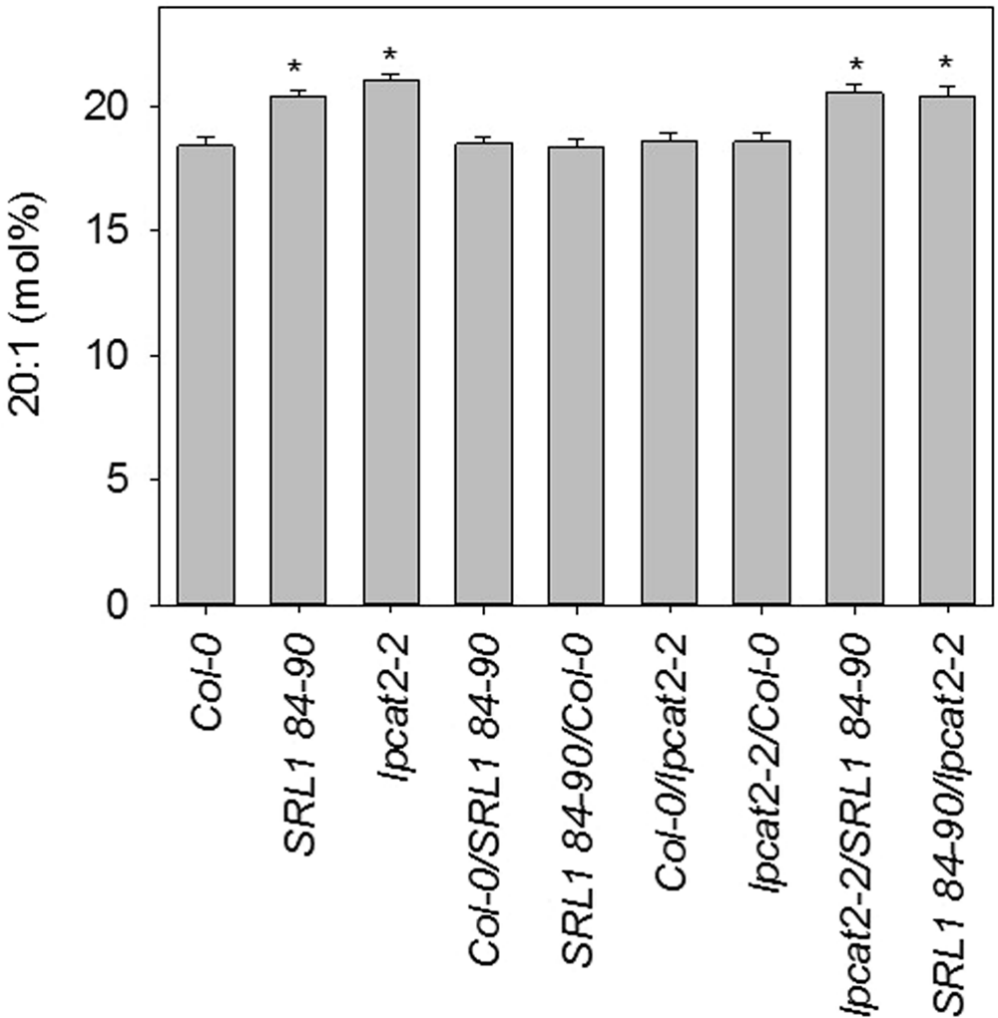
Complementation test between 20:1q2 near isogenic line SRL1 84–90 and *lpcat2-2*. Reciprocal crosses were performed between Col-0, *lpcat2-2* and SRL1 84–90, carrying a single Ler-0 introgression in the region of 20:1q2. The fatty acid composition of heterozygous (and homozygous) F1 seed from five plants of each genotype (*n* = 5) was measured and 20:1 content is the mean ± s.e.m. The asterisk denotes values that are significantly different (*P* > 0.05) from Col-0 (LSD-test). For heterozygous F1 seed the maternal parent is listed first.

To test whether variation in *LPCAT2* allele function between Col-0 and Ler-0 is sufficient to explain 20:1q2, we performed a complementation test^15^. We carried out reciprocal crosses between wild-type Col-0, the homozygous *lpcat2-2* mutant^32^ and SRL1 84*-*90, which carries a single Ler-0 introgression at 20:1q2. We then analysed the fatty acid composition of heterozygous F1 seed and self-pollinated homozygous F1 seed (Fig. 6). The 20:1 content of Col-0/SRL1 84–90 and Col-0/*lpcat2-2* seed was not significantly different from Col-0 (*P* < 0.05), suggesting that *lpcat2-2* and Ler-0 20:1q2 are both recessive hypomorphic mutant alleles (Fig. 6). When we measured the 20:1 content of *lpcat2-2*/SRL1 84–90 seed it was significantly higher than Col-0 (*P* < 0.05) (Fig. 6). This lack of complementation by recessive alleles suggests that *LPCAT2* and 20:1q2 are synonymous.

### An INDEL in the *LPCAT2* promoter is responsible for the variation in seed 20:1 content

**Figure 7:**
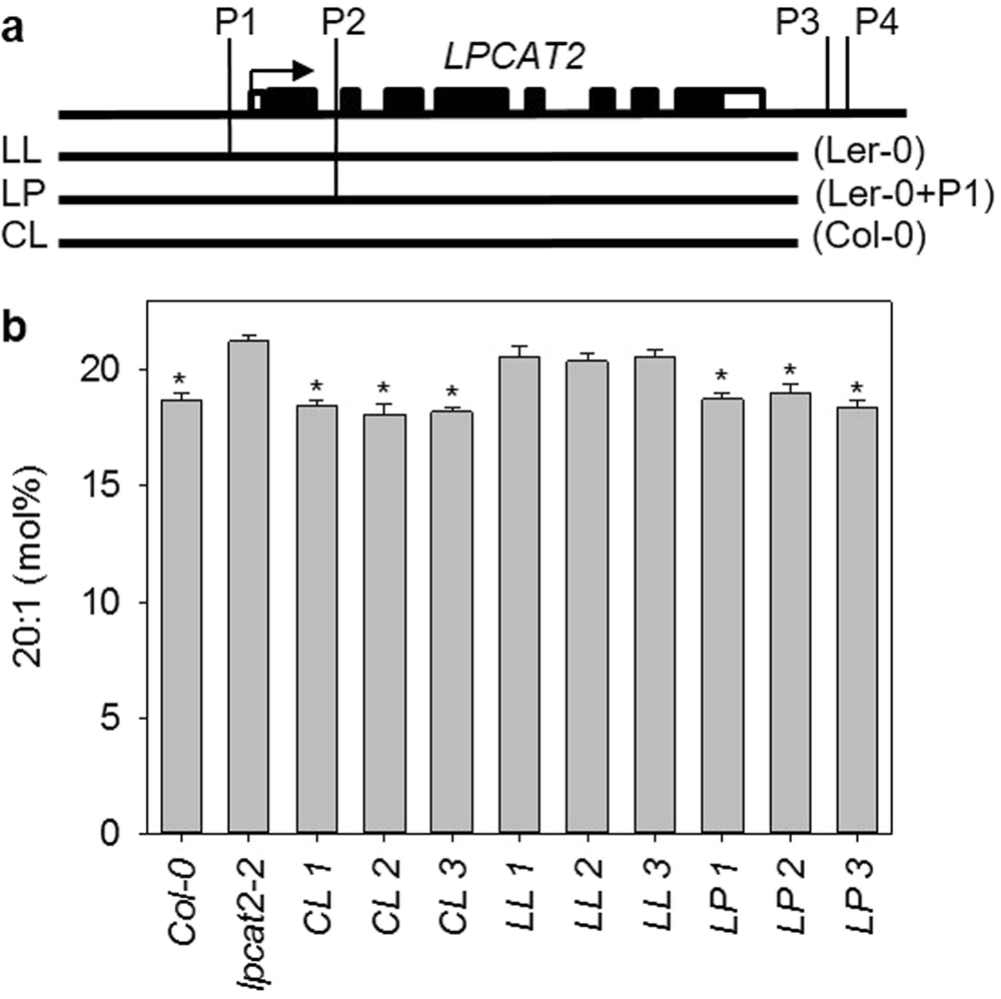
Identification of the polymorphism that causes variation in *LPCAT2* function between Col-0 and Ler-0 alleles. (**a**) T-DNA constructs containing a ~3.3 kb genomic region of Col-0 *LPCAT2* (CL), Ler-0 *LPCAT2* (LL) or LL with a Col-0 variant of P1 (LP) were transformed into *lpcat2-2*. (**b**) Fatty acid analysis was performed on seeds from three homozygous transformants (1–3) for each construct and 20:1 content is the mean ± s.e.m. of measurements on five plants (*n* = 5) of each genotype. The asterisk denotes values that are not significantly different (*P* < 0.05) from Col-0 (LSD-test).

Four polymorphisms in *LPCAT2* are strongly associated with both seed 20:1 content and *LPCAT2* expression level in the MAGIC population (Fig. 2, Supplementary Table 3). These polymorphisms form a haplotype that distinguishes Col-0 from Ler-0 *LPCAT2*. P1 is an insertion-deletion (INDEL) that lies early in the promoter of *LPCAT2*, P2 is a single nucleotide polymorphism (SNP) in the first intron and P3 and P4 are SNPs that lie downstream in the 3′ intergenic region (Supplementary Table 3). To test whether one or more of these polymorphisms cause variation in *LPCAT2* function between Col-0 and Ler-0, we transformed *lpcat2-2* with three different T-DNA constructs (Fig. 7a). The first construct (CL) contained a ~3.3 kb genomic region of Col-0 *LPCAT2* encompassing P1 and P2. The second construct (LL) contained the corresponding genomic region of Ler-0 *LPCAT2*. Finally, the third construct (LP) was the same as LL, but contained the Col-0 variant of P1. Fatty acid analysis performed on seeds from three independent homozygous transgenic lines containing each construct showed that CL and LP could both complement the seed 20:1 content phenotype of *lpcat2-2*, whereas LL could not (Fig. 7b). Although we cannot rule out a contribution from other polymorphisms in the region of *LPCAT2*, our data suggest that P1 (a 3 bp INDEL at −27 bp in the promoter) is sufficient to explain allelic variation in *LPCAT2* function between Col-0 and Ler-0, and by extension 20:1q2 in the MAGIC population.

## Discussion

**Figure 8:**
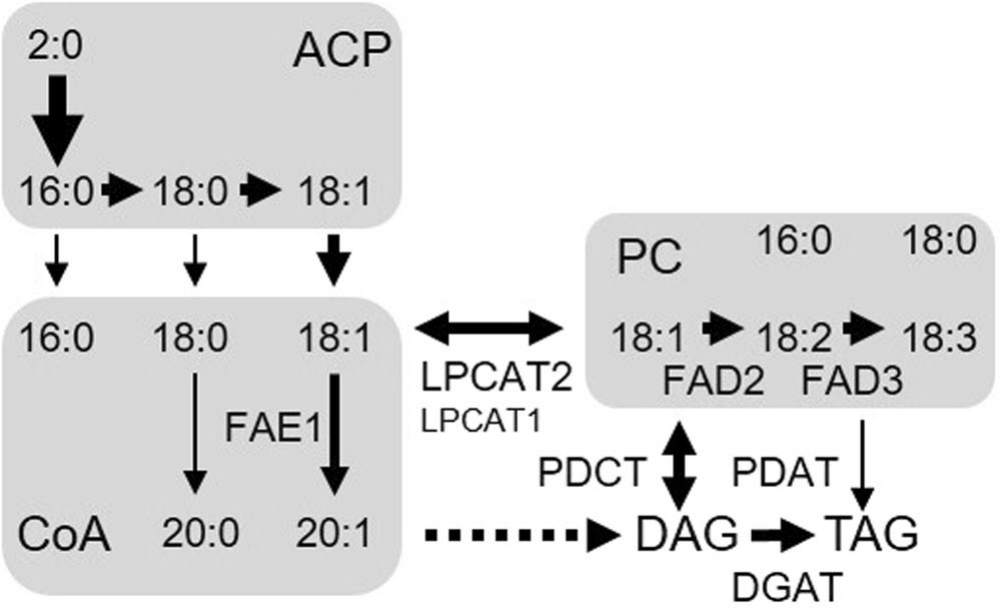
A simplified diagram of fatty acid synthesis, modification and assembly into triacylglycerol in developing *Arabidopsis* seeds^8^. The diagram illustrates the role of LPCAT in acyl partitioning between the cytosolic acyl-Coenzyme A (CoA) and phosphatidylcholine (PC) pools used for microsomal elongation and desaturation. Major acyl groups made (or present) in the plastidial acyl-acyl carrier protein (ACP) and cytosolic acyl-CoA and PC pools are shown. 2:0 is acetic acid; 16:0 is palmitic acid, 18:0 is stearic acid, 18:1 is oleic acid, 20:0 is eicosanoic acid, 20:1 is eicosenoic acid, 18:2 is linoleic acid and 18:3 is linolenic acid. DAG is diacylglycerol, TAG is triacylglycerol, LPCAT is acyl-CoA:lysophosphatidylcholine acyltransferase, FAE is fatty acid elongase, FAD is fatty acid desaturase, PDCT is PC:DAG cholinephosphotransferase, PDAT is phospholipid:DAG acyltransferase and DGAT is acyl-CoA:DAG acyltransferase. The dotted arrow represents the first three catalytic steps of the Kennedy pathway responsible for acyl-CoA dependent formation of DAG from glycerol-3-phosphate.

In this study, we show that natural variation in *LPCAT2*^31–33^ is a determinant of seed storage oil composition in *Arabidopsis*. LPCAT catalyses the reversible acylation of 1-LPC^34,35^, and in doing so enables acyl-exchange (or ‘acyl editing’) between the acyl-CoA and PC pools that are the respective sites of microsomal fatty acid elongation and desaturation^31,32^ (Fig. 8). Previous work has established that acyl-editing makes a major contribution to acyl flux into TAG in several oilseed species^31,32,39–42^ and disruption of *LPCAT* in *Arabidopsis* decreases fatty acid unsaturation and increases chain length^31,32^. There has long been speculation that LPCAT activity contributes to the regulation of seed TAG composition, particularly in PUFA-rich species^32,42^. However, acyl editing also takes place without any acyl modification^31,32,41,42^. Our study establishes that natural variation in acyl editing exists in oilseeds and is a factor that contributes to the genetic regulation of TAG composition.

Within the MAGIC population^24^, we found that a causal sequence variant in *LPCAT2* (P1) is a small INDEL situated in the promoter. *Arabidopsis* contains two *LPCAT* genes and Wang *et al*., (2012) showed that *LPCAT2* is the predominant form in developing seeds^32^. It is not known precisely how *LPCAT2* expression is regulated. However, initiation of transcription by RNA polymerase II requires assembly of a basal transcription apparatus at the core promoter, a region of ~70 bp flanking the transcription start site (TSS)^43^. P1 is situated just 27 bp upstream of the *LPCAT2* TSS^44^ (Supplementary Fig. 1) and it may therefore affect transcriptional initiation, either by modifying a recognition element^42^ or by changing promoter context^45^. Extensive *cis* regulation of gene expression is thought to exist in *Arabidopsis*^46^ and Gan *et al*.^25^ previously reported that potential *cis*-acting sequence variants, associated with differential gene expression in seedlings of the MAGIC founder accessions, are concentrated in the ~100 bp promoter region^25^. Bioinformatic analysis of 1,135 *Arabidopsis* genomes sequences from the 1001 Genomes Consortium^47^ suggests that P1 is not a rare allele. The hypermorphic Col-0 variant is present in ~55% of accessions. Furthermore, there is a significant relationship between the genotype at P1 and latitude at the accession collection site^47^ (Col-0 variant = 48.96° ± 0.29, *n* = 622 and Ler-0 variant = 47.43° ± 0.33, *n* = 510; *P* = 0.0003, two-tailed t-test). An increased capacity for acyl editing at higher latitude is consistent with a need for more substrate to support microsomal fatty acid desaturation al lower temperatures^28,48^. Hence, allelic variation at P1 may be significant in local adaptation to temperature in natural populations of *Arabidopsis*^48^.

Natural variation is seed TAG composition has been studied quite extensively in *Arabidopsis* and many QTL have previously been identified^6,14–16,18,27,28^. In several studies, the main-effect QTL for VLCFA (or 20:1) content has mapped to the location of *FAE1*^6,15,16^. The identification of *LPCAT2* as a main-effect QTL for 20:1 content in the MAGIC population was therefore surprising, but this just reflects a lack of causal allelic variation in *FAE1* within the 19 MAGIC founder accessions. These founder accessions are not polymorphic for the nonsynonymous causal sequence variant in *FAE1* that was identified by Jasinski *et al*., (2012) and no polymorphisms exist between Col-0 and Ler-0 within a region ~300 bp up and downstream of *FAE1*^6,25,37^. A minor QTL (20:1q3) in the MAGIC population did map to the approximate location of *FAE1*. However, we could not identify individual sequence variants that are significantly associated with 20:1 content within the 90% CI for 20:1q3.

We could identify individual sequence variants that are significantly associated with 20:1 content within the 90% CI of one other minor QTL (20:1q1). 20:1q1 corresponds approximately to the location of a 20:1/18:1 (oleic acid) ratio QTL previously identified by O’Neill *et al*. (2012) in a Wietze (Wt-5) × Catania (Ct-1) biparental mapping population^16^. Ct-1 is one of the 19 MAGIC founder accessions^24^. We found associated sequence variants within 1 kb up or downstream of several genes in the 20:1q1 90% CI, including three U-box containing proteins that are potential E3 ubiquitin ligases^49^, three transcription factors, a ATP-binding cassette transporter and a glycerol-3-phosphate acyltransferase (GPAT4) (Supplementary Data 1). Among these genes, only *GPAT4* has previously been ascribed a function in lipid metabolism. However, GPAT4 is unlikely to be 20:1q1 because it has been shown to have a specialised role in producing oxygenated *sn-2* acyl glycerol monomers for the extracellular polymer cutin^50,51^. It is noteworthy that 20:1q1 is located relatively close to *LPCAT1* and *lpcat1* seeds exhibit a slight increase in 20:1^32^. However, *LPCAT1* lies outside the 20:1q1 90% CI and no significantly associated sequence variants were detected near this gene (Fig. 1). Further work will be required to identify the causal polymorphism(s) for 20:1q1.

In conclusion, we have found that natural variation in a gene encoding the acyl editing enzyme LPCAT2 influences *Arabidopsis* seed TAG composition. LPCATs partition newly synthesised fatty acids between the acyl-CoA and PC substrate pools used for microsomal fatty acid elongation and desaturation, respectively^31,32^. Previous studies have identified natural variation in the enzymes that are directly responsible for fatty acid modification, such as FAE1^15^ and FATTY ACID DESATURASE 2^28^. *In vivo* pulse radiolabelling studies have shown that, in addition to acyl editing, PC-diacylglycerol (DAG) interconversion also makes a major contribution to acyl flux into TAG in several PUFA-rich oilseed species^42^ (Fig. 8). The main mechanism for PC-DAG interconversion in *Arabidopsis* is head group exchange, catalysed by phosphatidylcholine:diacylglycerol cholinephosphotransferase (PDCT)^31,52^. Phospholipid:diacylglycerol acyltransferase (PDAT) also transfers acyl groups to TAG directly from the *sn-2* position in PC^53^ (Fig. 8) and LPCAT2 is required to re-esterify the 1-LPC co-product^36^. It will therefore be interesting to explore whether natural variation also exists in PDCT and PDAT, which like LPCAT are not directly involved in acyl modification.

## Materials and Methods

### Plant material and growth conditions

The *Arabidopsis thaliana* MAGIC population founder accessions and recombinant inbred lines (RILs) (N782242) and the STAIRS single recombinant lines (SRLs) (N721831) were obtained from the European *Arabidopsis* Stock Centre (University of Nottingham, UK). The *lpcat2-2* mutant^32^ has been described previously. Seeds were sown on moist Levington F2 compost in P40 trays and vernalized for 6 weeks where necessary before being transferred to a controlled environment chamber or an air-conditioned glasshouse set to a 16-h light (22 °C)/8-h dark (16 °C) cycle. After one week seedlings were individually transplanted to 7 cm^2^ pots. For the initial analysis of the MAGIC RILs, the pots were arranged into a random block design in the glasshouse^28^. The plants were bagged individually at the onset of flowering^54^ and the seeds were then harvested at maturity.

### Analysis of seed fatty acid composition

The total fatty acid composition of seeds was measured by gas chromatography^55^, using the combined digestion and fatty acid methyl ester formation method^56^.

### Genetic analysis

Quantitative Trait Loci (QTL) mapping with the MAGIC population was carried out as described by Kover *et al*. (2009), using the ‘HAPPY R’ package from, http://archive.is/mus.well.ox.ac.uk ^24^. Genome-wide association studies (GWAS) were performed with the ‘magic_src_v4.0.tar.gz’ package, which can be obtained from the same site and includes detailed instructions. In brief, the ‘reconstruction’ program generates imputed genomes for the RILs with a mosaic breakpoint accuracy of >2 kb, using polymorphism calls derived from low coverage sequence and 1.2 M biallelic variants from the complete genomes of the 19 MAGIC founder accessions^26^. The ‘genome_scan’ program then performs association mapping, using all ~3 M individual sequence variants imputed from the 19 MAGIC founders. Specific sequence variants in the genomes of the 19 MAGIC founders were visualised with the Rätsch lab GBrowse tool (http://gbrowse.inf.ethz.ch/gb/gbrowse/thaliana-19magic). Sequence variants in 1,135 *Arabidopsis* genomes were visualised using Polymorph 1001 (http://tools.1001genomes.org/polymorph/).

### Gene expression analysis

RNA was purified from mature seeds and developing siliques and DNase-treated using the RNeasy kit from Qiagen with modifications described previously^57^. Single-stranded cDNA synthesis was performed using SuperScript II RNase H- reverse transcriptase from Invitrogen. A MyiQ Single-Color real-time PCR detection system (Bio-Rad) was used to carry out real-time PCR with the qPCR Mastermix Plus from Eurogentec. The data were analyzed using Bio-Rad iQ5, Optical System Software, version 2.0. The real-time PCR primer pairs were LPCAT2_Q (5′-tgcggttcagattccgcttttct-3′ and 5′-gttgccaccggtaaatagctttcg-3′) and 18S-Q (5′-tcctagtaagcgcgagtcatc-3′ and 5′- cgaacacttcaccggatcat-3′).

### Genotyping substitution lines

STAIRS single recombinant lines (SRLs) and the F2 progeny of a backcross to Col-0 were genotyped using PCR-based simple sequence length polymorphic markers^38^, as described by Koumproglou *et al*., (2002). In addition to using the existing markers nga 59, F20D23, nga 392, T27K12Sp6, nga 208, F5I14-49495 and nga 111^38^, we also created two new markers, using flanking INDELs situated ~1 kb 5′ and 3′ of the *LPCAT2* transcribed region. The primer pairs for PCR genotyping with these markers were LPCAT2_5′ (5′-aaaataacatgattttgagttgttgt3′ and 5′-ttgcaaataaatcataatatctaccaa-3′) and LPCAT2_3′ (5′-cgataaggcgctagatgctc-3′ and 5′-cacggcctctcttttcttctt-3′).

### Enzyme assay

Microsomal fractions were prepared from homogenates of ~1 g of developing siliques of each genotype and the acylation of lysophosphatidylcholine was then assayed following the methods described previously^32^.

### Cloning and plant transformation

A ~3.3 kb region of Col-0 and Ler-0 genomic DNA containing *LPCAT2* was amplified by PCR using primer pair (5′-ccacaggagggcgtcgaattttggtg-3′ and 5′-tggtccactcatcgtctcgctaatgt-3′) and cloned into the pENTR/D-TOPO vector. The Quikchange Lightning Site-Directed Mutagenesis Kit from Agilent Technologies (http://www.agilent.com) and primer pair (5′-aacttcacacaaacctcgtcaagatcgaaaccaaacccac-3′ and 5′-gtgggtttggtttcgatcttgacgaggtttgtgtgaagtt-3′) were then used to introduce the Col-0 variants of polymorphisms P1 (Supplementary Table 3) into the Ler-0 allele, following the manufacturer’s instructions. The gene cassettes were then cloned into the destination vector pEarleyGate301^58^ using the Gateway LR clonase enzyme mix from Invitrogen Ltd (http://www.thermofisher.com). Heat shock was used to transform the plasmids into *Agrobacterium tumefaciens* strain GV3101 and *Arabidopsis* transformation was then carried out using the floral-dip method^59^. Glufosinate resistance was used to select T0 primary transgenic lines and homozygous T3 lines were subsequently recovered and analysed.

### Statistical Analyses

The number of biological replicates (*n*) and the standard error of the mean (s.e.m.) are shown. ANOVA (one-way analysis of variance) was used to assess differences between genotypes for seed fatty acid measurements. Following significant (*P* < 0.05) F-test results, means were compared using the appropriate LSD (least significant difference) value at the 5% (*P* = 0.05) level of significance, on the corresponding df (degrees of freedom). These analyses were performed using GenStat (18th edition, VSN International Ltd, Hemel Hempstead, UK). Linear regression analysis was also performed using the function in SigmaPlot v14.0 (Systat Software Inc.).

## Electronic supplementary material

Supplementary Tables and Figures

Dataset 1

## Data Availability

The datasets generated during and/or analysed during the current study are available from the corresponding author on reasonable request.
