# Supplementary material for: Natural variation in acyl editing is a determinant of seed storage oil composition
Source: Sci Rep. 2018 Nov 26;8:17346. doi: 10.1038/s41598-018-35136-6 (PMC6255774; doi:10.1038/s41598-018-35136-6)
Supplement: Supplementary file 1 — Supplementary Tables and Figures [file 41598_2018_35136_MOESM1_ESM.pdf]

Natural variation in acyl editing is a determinant of seed storage oil composition.

Guillaume N. Menard[1], Fiona M. Bryant[1], Amelie A. Kelly[2], Christian P. Craddock[3], Irene Lavagi[4], Keywan Hassani-Pak[1], Smita Kurup[1], Peter J. Eastmond[1]

[1]Department of Plant Science, Rothamsted Research, Harpenden, Hertfordshire, AL5 2JQ, UK

[2]Georg-August-University, Albrecht-von-Haller-Institute for Plant Sciences, Justus-von-Liebig Weg 11, 37077 Göttingen, Germany

[3]Mt. San Jacinto College, Menifee Valley Campus, 28237 La Piedra Road, Menifee, CA 92584 USA

[4]Department of Microbiology and Plant Pathology, University of California Riverside, Riverside, CA 92521, USA

Supplementary Table 1. QTL detected for seed 20:1 content using the MAGIC population. Individual plants for 427 RILs were grown in three randomised blocks (*n* = 3) in the glasshouse and the seed for each plant harvested separately and analysed. QTL analysis was performed using the 'HAPPY R' package (Kover et al., 2009). logPmax = -log10 ANOVA *P*-value and ~90% CI = estimated 90% confidence interval. QTL with a genome-wide *P* value < 0.01 are shown and r^2 indicates the amount of variation explained by the QTL.

QTL	Chromosome	Peak (Mb)	logP_{max}	~90% CI (Mb)	*P* value	r^2
20:1q1	Chr1	0.6	5.21	~2.6	0.002	0.05
20:1q2	Chr1	23.3	15.84	~0.8	<0.001	0.22
20:1q3	Chr4	17.4	5.47	~2.5	0.002	0.06
20:1q4	Chr5	11.8	4.86	~2.8	0.005	0.03

Supplementary Table 2. Highest ranking 20:1q2 candidates based on GWAS score. Individual plants for 427 RILs from the MAGIC population were grown in three randomised blocks (n = 3) in the glasshouse. 20:1 content was derived from the total fatty acid composition of the mature seed and GWAS was performed using the 'magic_src_v4.0.tar.gz' package. Polymorphisms within the 20:1q2 90% CI that lie < 1 kb from a gene are ranked by highest –log10(p) score.

AGI	$-\log_{10}(p)$	Gene Name / Description
At1g63020	20.34	NUCLEAR RNA POLYMERASE D1A
At1g63005	20.19	MIR399B
At1g63120	20.16	RHOMBOID-LIKE 2
At1g62990	20.08	IRREGULAR XYLEM 11
At1g63000	20.00	NUCLEOTIDE-RHAMNOSE SYNTHASE/EPIMERASE-REDUCTASE 1
At1g63010	20.00	VACUOLAR PHOSPHATE TRANSPORTER 1
At1g63030	20.00	DWARF AND DELAYED FLOWERING 2
At1g63040	20.00	Pseudogene
At1g63050	20.00	LYSOPHOSPHATIDYLCHOLINE ACYLTRANSFERASE 2
At1g63060	20.00	Ribosome biogenesis NEP1-like protein
At1g63080	20.00	Transacting siRNA generating locus
At1g63090	20.00	PHLOEM PROTEIN 2-A11
At1g63100	20.00	Scarecrow-like protein 28
At1g63110	20.00	GPI transamidase subunit PIG-U
At1g62981	19.68	Transmembrane protein, putative
At1g62980	19.35	EXPANSIN A18
At1g63160	19.06	REPLICATION FACTOR C 2
At1g63180	19.06	UDP-D-GLUCOSE/UDP-D-GALACTOSE 4-EPIMERASE 3
At1g64410	19.05	Transposable element
At1g65050	18.74	TRAF-like superfamily protein

Supplementary Table 3. Polymorphisms within *LPCAT2* region that are associated with 20:1. Individual plants 427 RILs from the MAGIC population were grown in three randomised blocks (n = 3) in the glasshouse. 20:1 content was derived from analysis of the total fatty acid composition of the mature seed and GWAS was performed using the 'magic_src_v4.0.tar.gz' package. Polymorphisms are marked relative to the start of transcription in Col-0[43].

Name	Position	$-\log_{10}(p)$	Location	Type	Sequence
P1	Ch1:23378318	20.0	-27 bp (5' promoter)	INDEL	+TTG
P2	Ch1:23377804	20.0	+487 bp (1st intron)	SNP	T/A
P3	Ch1:23375800	20.0	+2491 bp (3' intergenic)	SNP	C/T
P4	Ch1:23375695	20.0	+2596 bp (3' intergenic)	SNP	A/G

Supplementary Figure 1. Sequence alignment of Col-0 and Ler-0 *LPCAT2*. Polymorphisms associated with seed 20:1 content (Supplementary Table 3) are marked red and others are marked grey. Translated and untranslated regions are marked green and yellow on the Col-0 sequence.

```
Col-0      tattacttcattaaaatttgttgctgtaattaatgaaattttgatatgaattaacaaata
Ler-0      TATTACTTCATTAAAATTTGTTGCTGTAATTAATGAAATTTTGATATGAATTAACAAATA
           ************************************************************

Col-0      catgaaaaaatgatctttcttataagaaagtttttgtagtatcttttattataatacaaa
Ler-0      CATGAAAAAATGATCTTTCTTATAAGAAAGTTTTTGTAGTATCTTTTATTATAATACAAA
           ************************************************************

Col-0      agttttgaaaacaaaattttatcaaatcccaaataaattttgattttgacataaggcgta
Ler-0      AGTTTTGAAAACAAAATTTTATCAAATCCCAAATAAATTTTGATTTTGACATAAGGCGTA
           ************************************************************

Col-0      cattaatattgattttgacaaaaggcgtaaaaaaaataaaaaaa----aaataaataaat
Ler-0      CATTAATATTGATTTTGACAAAAGGCGTAAAAAAAAAATAATAATAATAAATAAATAAAT
           ************************************ * ** **    ************

Col-0      aaattatattaacttcccatcaggcatcagaaatatagatcaaacattgagggtattatt
Ler-0      AAATTATATTAACTTCCCATCAGGCATCAGAAATATAGATCAAACATTGAGGGTATTATT
           ************************************************************

Col-0      gtaacttcacacaaacctcgtc---aagatcgaaaccaaacccacacgaactacaaaaga
Ler-0      GTAACTTCACACAAACCTCGTCAACAAGATCGAAACCAAACCCACACGAACTACAAAAGA
           **********************   ***********************************

Col-0      ccccatcagagagattccgattcagatgtccgaactgtgagagtcgtcgtcgtcgtcgta
Ler-0      CCCCATCAGAGAGATTCCGATTCAGATGTCCGAACTGTGAGAGTCGTCGTCGTCGTCGTA
           ************************************************************

Col-0      actcagtccgagttgacacaatcttccacttcacgcaagatacaaccATGGAATTGCTTG
Ler-0      ACTCAGTCCGAGTTGACACAATCTTCCACTTCACGCAAGATACAACCATGGAATTGCTTG
           ************************************************************

Col-0      ACATGAACTCAATGGCTGCCTCAATCGGCGTCTCCGTCGCCGTTCTCCGTTTCCTCCTCT
Ler-0      ACATGAACTCAATGGCTGCCTCAATCGGCGTCTCCGTCGCCGTTCTCCGTTTCCTCCTCT
           ************************************************************
```

```
Col-0       GTTTCGTCGCAACGATACCAATCTCATTTTTATGGCGATTCATCCCGAGTCGACTCGGTA
Ler-0       GTTTCGTCGCAACGATACCAATCTCATTTTTATGGCGATTCATCCCGAGTCGACTCGGTA
            ************************************************************
Col-0       AACACATATACTCAGCTGCTTCTGGAGCTTTCCTCTCTTATCTCTCCTTTGGCTTCTCCT
Ler-0       AACACATATACTCAGCTGCTTCTGGAGCTTTCCTCTCTTATCTCTCCTTTGGCTTCTCCT
            ************************************************************
Col-0       CAAATCTTCACTTCCTTGTCCCAATGACGATTGGTTACGCTTCAATGGCGATTTATCGAC
Ler-0       CAAATCTTCACTTCCTTGTCCCAATGACGATTGGTTACGCTTCAATGGCGATTTATCGAC
            ************************************************************
Col-0       CCTTGTCTGGATTCATTACTTTCTTCCTAGGCTTCGCTTATCTCATTGGCTGgtaaagct
Ler-0       CCTTGTCTGGATTCATTACTTTCTTCCTAGGCTTCGCTTATCTCATTGGCTGGTAAAGCT
            ************************************************************
Col-0       taaaactttatgctatatagtctctaaagcttgctccttttaatcgattttgtgattttg
Ler-0       TAAAACTTTATGCTATATAGTCTCTAAAGCTTGCTCCTTTTAATCGATTTTGTGATTTTG
            ************************************************************
Col-0       tatatgtagTCATGTGTTTTATATGAGTGGTGATGCTTGGAAAGAAGGAGGAATTGATTC
Ler-0       TTTATGTAGTCATGTGTTTTATATGAGTGGTGATGCTTGGAAAGAAGGAGGAATTGATTC
            * **********************************************************
Col-0       TACTGgtactctttttttctctcgatttgatggtttattattggtttatgttcttagaaga
Ler-0       TACTGGTACTCTTTTTTTCTCTCGATTTGATGGTTTATTATTGGTTTATGTTCTTAGAAGA
            ************************************************************
Col-0       gtgtgaagttagtaggaactattggaggctgaatcttgattgtgattatagGAGCTTTGA
Ler-0       GTGTGAAGTTAGTAGGAACTATTGGAGGCTGAATCTTGATTGTGATTATAGGAGCTTTGA
            ************************************************************
Col-0       TGGTATTAACACTGAAAGTGATTTCGTGTTCGATAAACTACAACGATGGAATGTTGAAAG
Ler-0       TGGTATTAACACTGAAAGTGATTTCGTGTTCGATAAACTACAACGATGGAATGTTGAAAG
            ************************************************************
Col-0       AAGAAGGTCTACGTGAGGCTCAGAAGAAGAACCGTTTGATTCAGATGCCTTCTCTTATTG
Ler-0       AAGAAGGTCTACGTGAGGCTCAGAAGAAGAACCGTTTGATTCAGATGCCTTCTCTTATTG
            ************************************************************
```

```
Col-0         AGTACTTTGGTTATTGCCTCTGTTGTGGAAGCCATTTCGCTGGCCCGGTTTTCGAAATGA
Ler-0         AGTACTTTGGTTATTGCCTCTGTTGTGGAAGCCATTTCGCTGGCCCGGTTTTCGAAATGA
              ************************************************************
Col-0         AAGATTATCTCGAATGGACTGAAGAGAAAGGAgtaagtgtttttctctctcgttcttcgg
Ler-0         AAGATTATCTCGAATGGACTGAAGAGAAAGGAGTAAGTGTTTTTCTCTCTCGTTCTTCGG
              ************************************************************
Col-0         tctatgtacttgtcgaaaaagtgaggttcttgattgtcttgtgtgtgggtttcagATTTG
Ler-0         TCTATGTACTTGTCGAAAAAGTGAGGTTCTTGATTGTCTTGTGTGTGGGTTTCAGATTTG
              ************************************************************
Col-0         GGCTGTTTCTGAAAAAGGAAAGAGACCATCGCCTTATGGAGCAATGATTCGAGCTGTGTT
Ler-0         GGCTGTTTCTGAAAAAGGAAAGAGACCATCGCCTTATGGAGCAATGATTCGAGCTGTGTT
              ************************************************************
Col-0         TCAAGCTGCGATTTGTATGGCTCTCTATCTCTATTTAGTACCTCAGTTTCCGTTAACTCG
Ler-0         TCAAGCTGCGATTTGTATGGCTCTCTATCTCTATTTAGTACCTCAGTTTCCGTTAACTCG
              ************************************************************
Col-0         GTTCACTGAACCAGTGTACCAAGAATGGGGATTCTTGAAGAGATTTGGTTACCAATACAT
Ler-0         GTTCACTGAACCAGTGTACCAAGAATGGGGATTCTTGAAGAGATTTGGTTACCAATACAT
              ************************************************************
Col-0         GGCGGGTTTCACGGCTCGTTGGAAGTATTACTTTATATGGTCTATCTCAGAGGCTTCTAT
Ler-0         GGCGGGTTTCACGGCTCGTTGGAAGTATTACTTTATATGGTCTATCTCAGAGGCTTCTAT
              ************************************************************
Col-0         TATTATCTCTGGTTTGGGTTTCAGTGGTTGGACTGATGAAACTCAGACAAAGGCTAAATG
Ler-0         TATTATCTCTGGTTTGGGTTTCAGTGGTTGGACTGATGAAACTCAGACAAAGGCTAAATG
              ************************************************************
Col-0         GGACCGCGCTAAGAATGTCGATATTTTGGGGGTTGAGCTTGCCAAGAGTGCGGTTCAGAT
Ler-0         GGACCGCGCTAAGAATGTCGATATTTTGGGGGTTGAGCTTGCCAAGAGTGCGGTTCAGAT
              ************************************************************
Col-0         TCCGCTTTTCTGGAACATACAAGTCAGCACATGGCTCCGTCACTgtgagtaatgatgctt
Ler-0         TCCGCTTTTCTGGAACATACAAGTCAGCACATGGCTCCGTCACTGTGAGTAATGATGCTT
              ************************************************************
```

```
Col-0     atgaggagatcctagagattgccatatcttataagtcttttaaccgtttcttcttattga
Ler-0     ATGAGGAGATCCTAGAGATTGCCATATCTTATAAGTCTTTTAACCGTTTCTTCTTATTGA
          ************************************************************
Col-0     tgcagACGTATATGAGAGAATTGTGAAGCCCGGGAAGAAAGCGGGTTTCTTCCAATTGCT
Ler-0     TGCAGACGTATATGAGAGAATTGTGAAGCCCGGGAAGAAAGCGGGTTTCTTCCAATTGCT
          ************************************************************
Col-0     AGCTACGCAAACCGTCAGTGCTGTCTGGCATgtgagtgctccttcccttctttgctatac
Ler-0     AGCTACGCAAACCGTCAGTGCTGTCTGGCATGTGAGTGCTCCTTCCCTTCTTTGCTATAC
          ************************************************************
Col-0     tctcagctaagatttggtttgagaagtcttgaaaagtcgcggttctttattgtttacggt
Ler-0     TCTCAGCTAAGATTTGGTTTGAGAAGTCTTGAAAAGTCGCGGTTCTTTATTGTTTACGGT
          ************************************************************
Col-0     gataaaggaatcatttttacatgtccgcactgcgaattctgcagaaagattcaatctttg
Ler-0     GATAAAGGAATCATTTTTACATGTCCGCACTGCGAATTCTGCAGAAAGATTCAATCTTTG
          ************************************************************
Col-0     tgtgctaaagaaagtaattaaaacctctacttgtgtttttatttgcagGGACTGTATCCT
Ler-0     TGTGCTAAAGAAAGTAATTAAAACCTCTACTTGTGTTTTTATTTGCAGGGACTGTATCCT
          ************************************************************
Col-0     GGATACATTATATTCTTTGTGCAATCAGCATTGATGATCGATGGTTCGAAAGgtacgata
Ler-0     GGATACATTATATTCTTTGTGCAATCAGCATTGATGATCGATGGTTCGAAAGGTACGATA
          ************************************************************
Col-0     ctagccttcatctttcatttaatgttctgttttgattcagtttcagtaaataagtaatct
Ler-0     CTAGCCTTCATCTTTCATTTAATGTTCTGTTTTGATTCAGTTTCAGTAAATAAGTAATCT
          ************************************************************
Col-0     aaaaacttctccctttgtattaaacagCTATTTACCGGTGGCAACAAGCAATACCTCCGA
Ler-0     AAAAACTTCTCCCTTTGTATTAAACAGCTATTTACCGGTGGCAACAAGCAATACCTCCGA
          ************************************************************
Col-0     AAATGGCAATGCTGAGAAATGTTTTGGTTCTCATCAATTTCCTCTACACAGTAGTGGTTC
Ler-0     AAATGGCAATGCTGAGAAATGTTTTGGTTCTCATCAATTTCCTCTACACAGTAGTGGTTC
          ************************************************************
```

```
Col-0      TCAATTACTCATCCGTCGGTTTCATGgtaactaaaaactcttccctccgtttttttcaac
Ler-0      TCAATTACTCATCCGTCGGTTTCATGGTAACTAAAAACTCTTCCCTCCGTTTTTTTCAAC
           ************************************************************

Col-0      ggtgttttcatttcaaaaaactctcatttttgtgtgtaatttttcagGTTTTAAGCTTGC
Ler-0      GGTGTTTTCATTTCAAAAAACTCTCATTTTTGTGTGTAATTTTTCAGGTTTTAAGCTTGC
           ************************************************************

Col-0      ACGAAACACTAGTCGCCTTCAAGAGTGTATATTACATTGGAACAGTTATACCTATCGCTG
Ler-0      ACGAAACACTAGTCGCCTTCAAGAGTGTATATTACATTGGAACAGTTATACCTATCGCTG
           ************************************************************

Col-0      TGCTTCTTCTCAGCTACTTAGTTCCTGTGAAGCCTGTTAGACCAAAGACCAGAAAAGAAG
Ler-0      TGCTTCTTCTCAGCTACTTAGTTCCTGTGAAGCCTGTTAGACCAAAGACCAGAAAAGAAG
           ************************************************************

Col-0      AATAAtgttgtctttttaaaaaatcaacaacattttggttcttttctttttttccacttg
Ler-0      AATAATGTTGTCTTTTTAAAAAATCAACAACATTTTGGTTCTTTTCTTTTTTTCCACTTG
           ************************************************************

Col-0      gaccgttttatgtaaaacaagagaaatcaagatttgaggttttattcttcttctccttcc
Ler-0      GACCGTTTTATGTAAAACAAGAGAAATCAAGATTTGAGGTTTTATTCTTCTTCTTCTTCC
           ****************************************************** *****

Col-0      caattttcgaaaatgattttattttttctgatatatatctaagctagtccaaa----gtc
Ler-0      CAATTTTCGAAAATGATTTTATTTTTTCTGATATATATCTAAGCTAGTCCAAATTAAGTC
           *****************************************************    ***

Col-0      aactcgaaaatgaacaaaaaggattaaaaagttcatatattttttggttgaaagaggtca
Ler-0      AACTCGAAAATGAACAAAAAGGATTAAAAAGTTCATATATTTTTTGGTTGAAAGAGGTCA
           ************************************************************

Col-0      agtttattcgaatctgaatttcttttaacggaacaacaagtgtgacaaaacaagattctt
Ler-0      AGTTTATTCGAATCTGAATTTCTTTTAACAGAACAACAAGTGTGACAAAACAAGATTCTT
           ***************************** ******************************

Col-0      ctccggcac
Ler-0      CTCCAGCAC
           **** ****
```
